# Bladder paraganglioma: a 25-year systematic review unveils the benefits of early diagnosis in reducing surgical complications

**DOI:** 10.3389/fsurg.2025.1657833

**Published:** 2025-09-22

**Authors:** Qingqing Liu, Tong Wang, Wenlong Tu, Pengfei Zhou, Xionghui Wu, Huayan Lv

**Affiliations:** ^1^ Department of Anesthesiology, Affiliated Jinhua Hospital, Zhejiang University School of Medicine (Jinhua Municipal Central Hospital), Jinhua, China; ^2^ Department of Biology, Duke University, Durham, NC, the United States; ^3^ Department of Urology, Affiliated Jinhua Hospital, Zhejiang University School of Medicine (Jinhua Municipal Central Hospital), Jinhua, China

**Keywords:** bladder paraganglioma, preoperative correct diagnosis, misdiagnosis, surgical complications, systematic review

## Abstract

**Background:**

Bladder paraganglioma (BPG) is a rare tumor frequently misdiagnosed before surgery. To raise awareness and improve early detection, we systematically reviewed clinical manifestations and treatment approaches reported in BPG case reports over the past 25 years. We aimed to identify factors that could facilitate timely diagnosis and reduce complications from misdiagnosis.

**Materials and methods:**

We searched PubMed for BPG case reports published between January 1, 2000, and December 31, 2024. Two independent reviewers extracted data and performed statistical analyses. Patients were categorized into two groups based on preoperative diagnosis: correctly diagnosed and misdiagnosed.

**Results:**

A total of 199 cases from 184 articles were included, with eighty patients (40.2%) preoperatively diagnosed with BPG and 119 (59.8%) misdiagnosed. Catecholamine-related symptoms were significantly more common in the correctly diagnosed group (60.0% vs. 21.8%, *p* < 0.001), whereas urinary symptoms were more prevalent in the misdiagnosed group (57.1% vs. 28.7%, *p* < 0.001). None of the misdiagnosed patients received preoperative *α*-adrenergic blockade therapy. There were significant differences in surgical approach selection between the two groups: 79.0% of patients in the misdiagnosed group underwent transurethral resection (TUR), while only 14.3% received partial cystectomy; conversely, in the correctly diagnosed group, 77.5% of patients underwent partial cystectomy, and only 15.0% underwent TUR. The correctly diagnosed group had more preoperative preparation (63.7% vs. 0%, *p* < 0.001), and lower incidences of intraoperative hypertensive crisis (17.5% vs. 40.3%, *p* < 0.001), discontinued surgeries (0% vs. 26.9%, *p* < 0.001), residual tumors (2.5% vs. 37.8%, *p* < 0.001), and reoperations (12.5% vs. 41.2%, *p* < 0.001). Catecholamine-related symptoms (OR = 3.98, *p* < 0.001) and hypertension (OR = 2.52, *p* = 0.015) predicted correct diagnosis while urinary symptoms (OR = 0.44, *p* = 0.031) were associated with misdiagnosis.

**Conclusion:**

More than half of BPG patients lack accurate preoperative diagnoses. Catecholamine-related symptoms were strongly associated with correct diagnosis, while urinary symptoms increased misdiagnosis. Patients with preoperative correct diagnosis more frequently received *α*-adrenergic blockade and underwent cystectomy rather than transurethral resection, with reduced hypertensive crises, surgery discontinuation, and residual tumors compared with those misdiagnosed. These findings highlight the need for structured catecholamine screening in bladder mass diagnostics and support our diagnostic flowchart to enhance early BPG detection.

## 1 Introduction

Pheochromocytoma and paragangliomas (PPGL) are rare neuroendocrine tumors, with annual prevalence about 1.4–6.6 per million person (1). Bladder paraganglioma (BPG) is one of the rarest types of thoracoabdominal paragangliomas, accounting for 0.7% of all PPGLs and 0.05% of all bladder tumors (2). Approximately 61.3% of BPGs are functional, secreting catecholamines and BPG symptoms arise both from tumor presence and catecholamine secretion, leading to a broad, nonspecific clinical spectrum including hematuria (47.2%), hypertension (54.7%), headache (48.1%), and syncope or palpitations (43.4%) (3). About 10% of functional tumors remain hormonally silent during daily activities but may provoke hypertensive crises during surgery due to catecholamine release (4). Generally, screening for BPG is recommended in the differential diagnosis of bladder masses, when some features of catecholamine excess or the classic “micturition attack” are present. For first-line screening, measurements of plasma or urinary free normetanephrine and metanephrine are recommended; while normal biochemical results could not entirely rule out BPG. All values above the reference range should be further investigated, while a value at least two-fold above the upper limit of normal (ULN) indicates a high probability of PPGL (5). Accurate preoperative diagnosis is challenging due to the absence of a definitive predictor.

This diagnostic difficulty elevates risks of missed or incorrect diagnosis, resulting in insufficient preoperative evaluation and preparation, intraoperative hypertensive crises, and subsequent complications. Hypertensive episodes can be prolonged and life-threatening, particularly when invasive hemodynamic monitoring and adequate antihypertensive treatments are lacking. Recent reports underscore the frequency and severity of intraoperative hypertensive crises in BPG, with considerable risk for acute heart failure and acute respiratory distress syndrome (6–8). Thus, preoperative identification, adequate preparation, and intraoperative vigilance are crucial for optimal outcomes. However, there are few specific guidelines for the diagnosis and management of BPG, and only marginally mentioned in guidelines or clinical practice consensus for PPGLs.

Since the first documented BPG case in 1953 by Zimmerman, numerous case reports and series have described initial misdiagnoses, severe intraoperative blood pressure fluctuations, and postoperative complications, but there is no specific systematic diagnostic algorithm as a basis (9). Although there are some systematic reviews of BPG, they are of limited help to clinicians in diagnosing BPG due to small sample sizes, lack of misdiagnosis analyses, and lack of a systematic diagnostic algorithm specifically for BPG (10). To better characterize BPG clinical features, diagnostic workup, pheochromocytoma crises, intraoperative management, and complications, we conducted a systematic review of BPG cases reported in PubMed over the past 25 years. By evaluating variables such as age, sex, tumor size, clinical presentations, presurgical diagnostic procedures, intraoperative events, and treatment modalities, this study will determine the predictors for preoperative diagnosis and develop a diagnostic and perioperative multidisciplinary management protocol to improve the preoperative diagnosis and reduce the misdiagnosis-associated perioperative complications. We hypothesized that patients with catecholamine-related symptoms would be more likely to receive a correct preoperative diagnosis, thereby reducing operative complications.

## 2 Materials and methods

### 2.1 Data sources and search strategy

Two investigators (QL and HL) independently screened English-language BPG case reports in PubMed (http://www.ncbi.nlm.nih.gov/pubmed) using the following search terms: [(pheochromocytoma (MeSH Terms)] OR [pheochromocytoma (Title/Abstract)] OR [paraganglioma (Title/Abstract)] OR [paraganglioma (MeSH Terms)]) AND ([bladder (MeSH Terms)] OR [bladder (Title/Abstract)]). The search was limited to publications between January 1, 2000, and December 31, 2024. No ethics approval required since this was a review of published literature. This study was guided by the PRISMA guideline (11).

### 2.2 Study selection and data analysis

Titles, abstracts, and full texts were independently reviewed by QL and HL. Cases involving patients under 18 years of age, patients who did not undergo surgery (cystoscopy, ultrasound-guided needle biopsy, and extravesical biopsy were not considered surgery) or lacking clear information on the timing of BPG diagnosis were excluded. Diagnosis required confirmation by pathology and immunohistochemistry. Extracted data included demographics, tumor size, mode of detection, presenting symptoms, catecholamine levels, hypertensive status, preoperative preparation, intraoperative hypertensive crises, biochemical and treatment methods.

During data extraction, we attempted to capture the sample type (plasma/urine) and specific metabolites (e.g., epinephrine/norepinephrine, metanephrine/normetanephrine) involved in catecholamine testing; however, such details were inconsistently and incompletely reported across included studies—some only noted “elevated plasma/urinary catecholamines” without specifying metabolites, while others reported single metabolites (e.g., norepinephrine) without additional data. This inconsistency precluded standardized integration of these variables for quantitative analysis.

Plasma and urine metanephrine/catecholamine levels were therefore categorized as normal, 1–3 times, or ≥3 times ULN. Levels above normal were considered indicative of catecholamine excess.

Patients were assigned to the correctly diagnosed group if diagnosed before initial surgery and to the misdiagnosed group if diagnosed postoperatively. We compared clinical features, presurgical workup, intraoperative complications, and treatment outcomes between groups.

Definitions included age at diagnosis and largest tumor diameter measured by CT or ultrasound (if CT unavailable). Mode of discovery was classified as incidental imaging detection, catecholamine-related symptoms (e.g., sweating, headache, palpitations, nausea/vomiting, intraoperative hypertensive crisis), urinary symptoms (e.g., hematuria, dysuria, pelvic pain), other evaluations, or unknown. Plasma and urine metanephrine/catecholamine levels were categorized as normal, 1–3 times, or ≥3 times ULN. Levels above normal were considered indicative of catecholamine excess. Functional BPG was defined by elevated biochemical markers, or intraoperative hypertensive episodes excluding other causes. Preoperative preparation was defined as the administration of *α*-adrenergic blockade before surgery. Hypertensive crisis was defined as systolic blood pressure exceeding 180 mmHg intraoperatively or significant blood pressure fluctuations. Cardiovascular complications included arrhythmias, acute heart failure, pulmonary edema, acute respiratory distress syndrome, Takotsubo syndrome, myocardial infarction, hypertensive encephalopathy, stroke, and aortic dissection. Metastatic PPGL was defined based on the World Health Organization (WHO) criteria as PPGL at non-chromaffin sites (bone, lymph nodes, liver) (12).

### 2.3 Statistical analysis

Data were analyzed using IBM SPSS Statistics 27. Normally distributed continuous variables were presented as mean ± standard deviation (Mean ± SD), non-normally distributed variables as median and interquartile range (Median, IQR). Group comparisons utilized *t*-test for normal data and Mann–Whitney *U* test for non-normal data. Categorical variables were expressed as counts and percentages and compared with Chi-square or Fisher's exact tests. Univariate and multivariate logistic regression were used to identify variables associated with correct preoperative diagnosis, with odds ratios (ORs) and 95% confidence intervals (CIs) reported. Statistical significance was defined as *p* < 0.05.

## 3 Results

### 3.1 Literature search results

**Figure 1:**
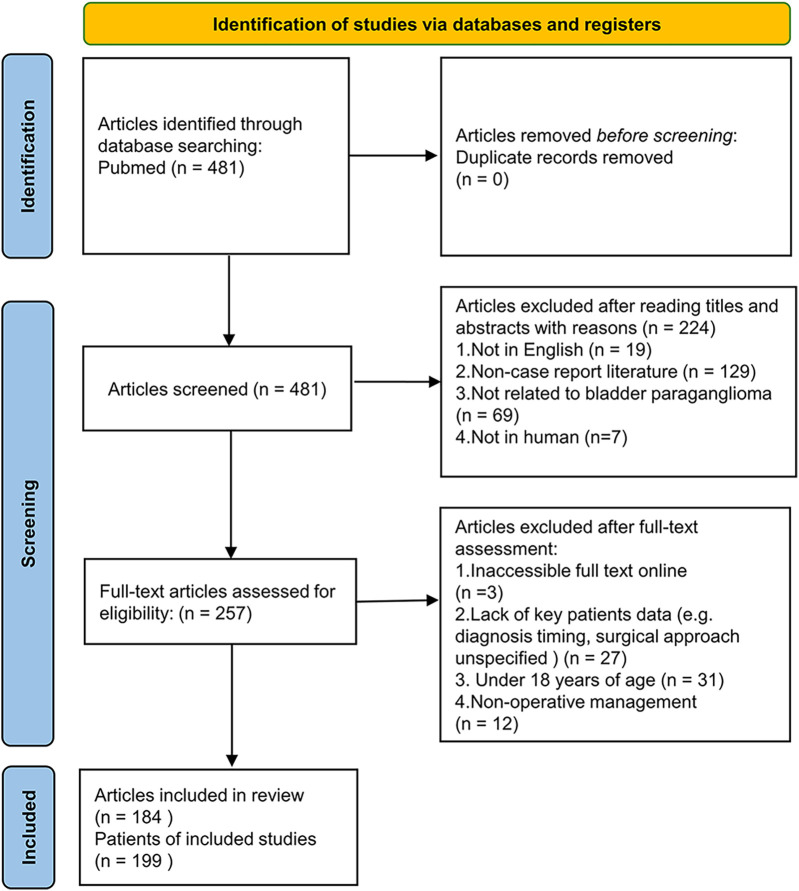
Flow diagram of the literature search and selection process. This figure illustrates the step-by-step inclusion and exclusion of studies in the systematic review. A total of 481 articles were initially identified, with 199 cases from 184 articles ultimately included in the pooled analysis based on predefined eligibility criteria.

A total of 481 articles were identified in PubMed (Figure 1). Articles were excluded for the following reasons: full-text papers unavailable (*n* = 3); not case reports (*n* = 129); not related to BPG (*n* = 69); lacking extractable detailed information on pre- or postsurgical diagnosis (*n* = 27); patients under 18 years of age (*n* = 31); patients who did not undergo surgery (*n* = 12); articles not in English (*n* = 19); and non-human studies (*n* = 7). After excluding 297 articles, 199 cases from 184 articles were included in the final analysis (Figure 1).

### 3.2 General characteristics of patients with BPG

We identified 199 patients diagnosed with BPG, including 114 women (57.3%). 80 (40.2%) of these were diagnosed with BPG before the first surgery, while 119 (59.8%) were diagnosed postoperatively. The mean age was 47.8 years (range, 18–81 years), and the average tumor size was 3.6 cm (range, 0.6–9.5 cm). Patients correctly diagnosed before surgery were younger than those misdiagnosed (45.0 ± 15.2 vs. 49.7 ± 16.4 years, *p* = 0.042) and had larger tumors (4.2 ± 2.0 vs. 3.3 ± 1.8 cm, *p* = 0.002). The correctly diagnosed group also had a higher proportion of tumor metastasis than the misdiagnosed group (17.5% vs. 7.6%, *p* = 0.032) (Table 1).

**Table 1:** Patients characteristics and clinical features of two groups.

Variables	Total	Correctly diagnosed	Misdiagnosed	*p*
Patients, *n*	199	80	119	
Age, mean ± SD (years)	47.8 ± 16.1	45.0 ± 15.2	49.7 ± 16.4	0.042
Female, *n* (%)	114 (57.3)	47 (58.7)	67 (56.3)	0.732
Tumor size, mean ± SD (cm)	3.6 ± 1.9	4.2 ± 2.0	3.3 ± 1.8	0.002
Symptoms, *n* (%)				
Incidental on imaging	35 (17.6)	11 (13.8)	24 (20.2)	0.244
Catecholamine-related symptoms	74 (37.2)	48 (60)	26 (21.8)	<0.001
Urinary symptoms	91 (45.7)	23 (28.7)	68 (57.1)	<0.001
Other	27 (13.6)	7 (8.8)	20 (16.8)	0.104
None	25 (12.6)	6 (7.5)	19 (16.0)	0.077
Micturition-triggered symptoms, *n* (%)	63 (31.7)	41 (51.2)	22 (18.5)	<0.001
Hypertension, *n* (%)	96 (48.2)	54 (67.5)	42 (35.3)	<0.001
Metastasis, *n* (%)	23 (11.6)	14 (17.5)	9 (7.6)	0.032

Values are expressed as mean ± SD or *n* (%) unless otherwise noted. *p* values were estimated by *t*-test for continuous variables and Chi-square test for categorical variables.

### 3.3 Clinical features of patients with BPG

**Figure 2:**
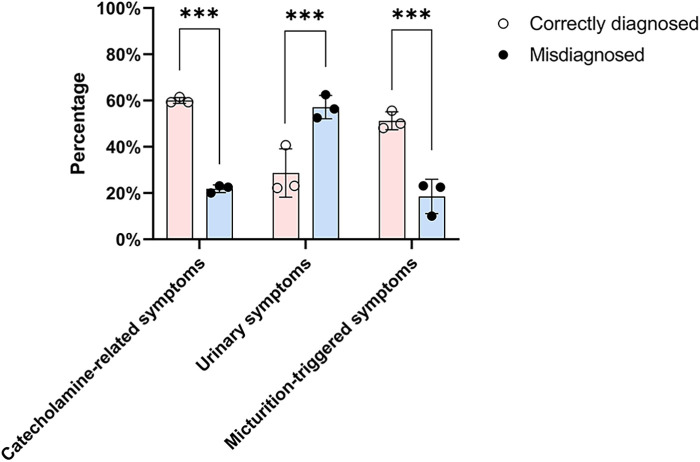
Distribution of presenting clinical symptoms among correctly diagnosed and misdiagnosed patients. The bar chart compares the frequency of catecholamine-related symptoms, urinary symptoms, and micturition-triggered events between the two groups. Chi-square test was used for comparison between the two groups. Correctly diagnosed patients more often presented with catecholamine-related or micturition-triggered symptoms, while urinary symptoms were more common among misdiagnosed patients (*** indicates statistical significance at *p* < 0.001).

Tumors were incidentally discovered on imaging in 35 patients (17.6%), identified due to catecholamine-related symptoms in 74 patients (37.2%), detected during evaluation for urinary symptoms (including hematuria, dysuria, pelvic pain, etc.) in 91 patients (45.7%), found during evaluation for other reasons in 27 patients (13.6%), or were of unknown origin in 25 patients (12.6%). The proportions of patients presenting with catecholamine-related symptoms and micturition-triggered catecholamine symptoms were significantly higher in the correctly diagnosed group than in the misdiagnosed group (60% vs. 21.8% and 51.2% vs. 18.5%, respectively; *p* < 0.001). Conversely, urinary symptoms were more common in the misdiagnosed group than in the correctly diagnosed group (57.1% vs. 28.7%, *p* < 0.001) (Table 1) (Figure 2). Although 74 patients reported catecholamine-related symptoms, only 48 (64.9%) were diagnosed with BPG prior to surgery. Among the 199 patients, 63 reported micturition-triggered catecholamine symptoms; of these, 41 were diagnosed presurgically, while 22 patients were initially overlooked. Notably, 12 of the overlooked patients reported only urinary symptoms preoperatively but retrospectively recalled hypertension, sudden palpitations, chest tightness, or headaches after urination upon diagnosis.

Hypertension history or new-onset hypertension was documented in 96 patients (48.2%). The frequency of hypertension was significantly higher in the correctly diagnosed group than in the misdiagnosed group (67.5% vs. 35.3%, *p* < 0.001) (Table 1). The classic triad of episodic hypertension, hematuria, and micturition-related attacks (such as post-micturition syncope) was rare, present in only 5 of 199 cases.

### 3.4 Diagnostic workup of patients with BPG

Biochemical evaluation for catecholamine excess was performed in 117 patients (58.8%). Among these, 70 underwent testing before surgery, and 47 after initial surgery. Correctly diagnosed patients were more likely to have undergone biochemical testing (87.5% vs. 39.5%, *p* < 0.001) (Table 2). Of those correctly diagnosed, 55 (78.5%) had BPG confirmed by biochemical evidence of catecholamine excess.

**Table 2:** Biochemical testing and image profile of two groups.

Variables	Total	Correctly diagnosed	Misdiagnosed	*p*
Biochemical testing, *n* (%)	117 (58.8)	70 (87.5)	47 (39.5)	<0.001
Result, *n* (%)				<0.001
≥3 times ULN	38 (19.1)	29 (36.3)	9 (7.5)	
1–3 times ULN	45 (22.6)	26 (32.5)	19 (16.0)	
Normal	34 (17.1)	15 (18.7)	19 (16.0)	
Unknown	82 (41.2)	10 (12.5)	72 (60.5)	
Imaging study performed, *n* (%)	83 (41.7)	45 (56.3)	38 (31.9)	<0.001
Positive imaging results, *n* (%)	64 (77.1)	37 (82.2)	27(71.1)	0.228

Values are expressed as *n* (%). *p* values were estimated by Chi-square test.

Imaging studies, including metaiodobenzylguanidine (MIBG) scintigraphy, positron emission tomography (PET), and F-18 fluorodeoxyglucose (FDG)-PET, were analyzed. In the correctly diagnosed group, 45 patients (56.3%) underwent at least one of these imaging modalities preoperatively, with 37 (82.2%) showing positive results. In contrast, 38 patients (31.9%) in the misdiagnosed group underwent these imaging tests postoperatively, with 27 (71.1%) yielding positive findings (Table 2).

Based on the study results, for patients with bladder tumors who present with specific symptoms (i.e., catecholamine-related symptoms or micturition-triggered symptoms), further investigation is recommended to be initiated. Biochemical testing for catecholamine excess was the core single modality. It was implemented in 87.5% (70/80) of correctly diagnosed patients—significantly higher than the 39.5% (47/119) in the misdiagnosed group (*p* < 0.001)—and directly facilitated diagnosis in 78.5% (55/70) of these cases. Importantly, the combination of “biochemical testing + specific imaging” yielded the highest correct diagnosis rate.

### 3.5 Treatment modality and intraoperative complications of patients with BPG

**Figure 3:**
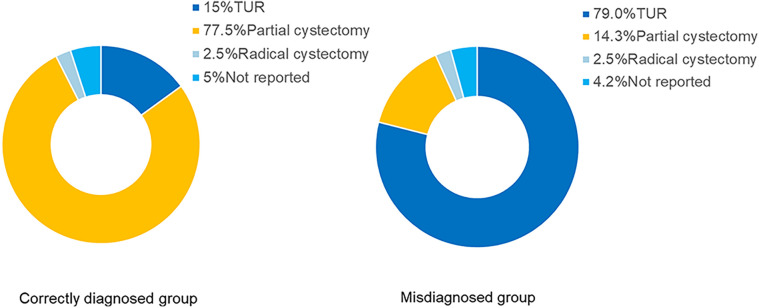
Initial surgical approaches in correctly diagnosed vs. misdiagnosed patients. This figure shows the distribution of transurethral resection, partial cystectomy, and radical cystectomy procedures across the two groups. Chi-square test was used for comparison between the two groups. Patients with correct preoperative diagnosis were more likely to undergo partial cystectomy, while those misdiagnosed commonly received transurethral resection (*p* < 0.001).

Initial surgical procedures were reported in 190 patients, 106 underwent transurethral resection (TUR), while 84 underwent partial or total cystectomy. There was a significant difference in surgical approach between groups: among correctly diagnosed patients, 62 (77.5%) underwent partial cystectomy, and 12 (15.0%) underwent TUR. In contrast, in misdiagnosed patients, most (94, 79.0%) underwent TUR, and only 17 (14.3%) had partial cystectomy (Table 3) (Figure 3).

**Table 3:** Surgical approaches and complications of two groups.

Variables	Total	Correctly diagnosed	Misdiagnosed	*p*
Preoperative preparation, *n* (%)	51 (25.6)	51 (63.7)	0 (0.0)	<0.001
Hypertensive crisis, *n* (%)	62 (31.2)	14 (17.5)	48 (40.3)	<0.001
Cardiovascular incidents during surgery, *n* (%)	5 (2.5)	0 (0.0)	5 (4.2)	0.084
Discontinued surgery, *n* (%)	32 (16.1)	0 (0.0)	32 (26.9)	<0.001
Reoperation, *n* (%)	59 (29.6)	10 (12.5)	49 (41.2)	<0.001
Residual tumor, *n* (%)	47 (23.6)	2 (2.5)	45 (37.8)	<0.001
Initial surgical approach, *n* (%)				<0.001
TUR	106 (53.3)	12 (15.0)	94 (79.0)	<0.001
Partial cystectomy	79 (39.7)	62 (77.5)	17 (14.3)	<0.001
Radical cystectomy	5 (2.5)	2 (2.5)	3 (2.5)	1.00
Not reported	9(4.5)	4(5.0)	5(4.2)	1.00

Values are expressed as *n* (%). *p* values were estimated by Chi-square test. TUR, transurethral resection.

**Figure 4:**
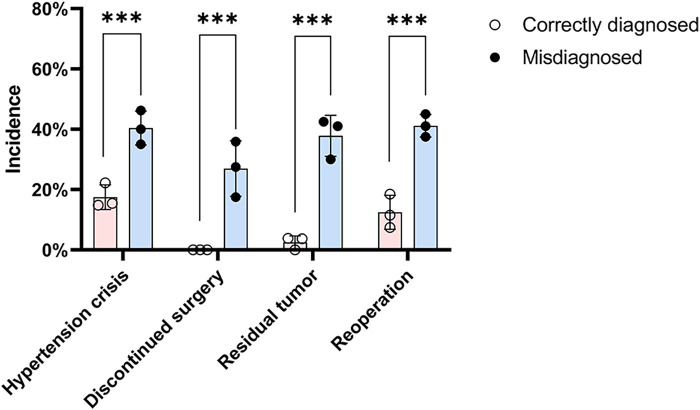
Incidence of intraoperative and postoperative complications in the two groups. The chart displays rates of intraoperative hypertensive crisis, discontinued surgery, residual tumor, and reoperation. Chi-square test was used for comparison between the two groups. Complication rates were significantly higher in the misdiagnosed group compared to those diagnosed preoperatively (*** indicates statistical significance at *p* < 0.001).

Preoperative *α*-adrenergic blockade was administered in 63.7% patients in the correctly diagnosed group, while none in the misdiagnosed group received such preparation (Table 3). Intraoperative hypertensive crisis occurred in 14 patients (17.5%) in the correctly diagnosed group, 11 of whom had received preoperative *α*-adrenergic blockade. Conversely, hypertensive crises occurred in 48 patients (40.3%) in the misdiagnosed group who had not received *α*-adrenergic blockade (*p* < 0.001) (Table 3) (Figure 4). No intraoperative cardiovascular incidents were reported among patients in the correctly diagnosed group; however, five patients in the misdiagnosed group experienced such events, all of whom lacked preoperative *α*-adrenergic blockade. Of all patients, at least 122 (≥61.3%) had functional paragangliomas, evidenced by elevated metanephrines or catecholamines in 83 patients and intraoperative hypertensive episodes in 39—considering some functional BPG patients without preoperative biochemical testing may also lack intraoperative hypertensive crises, as this is not inevitable.

Intraoperative hypertensive crisis occurred in 62 patients (31.2%), leading to surgery discontinuation in 32 (16.1%). Postoperative incomplete tumor removal was noted in 47 patients (23.6%) (Table 3). The correctly diagnosed group had fewer discontinued surgeries (0.0% vs. 26.9%, *p* < 0.001), less residual tumor (2.5% vs. 37.8%, *p* < 0.001), and fewer reoperations (12.5% vs. 41.2%, *p* < 0.001) than the misdiagnosed group (Table 3). Discontinued surgeries and residual tumor masses were significantly more frequent in the misdiagnosed group (Figure 4).

### 3.6 Catecholamine-related symptoms and hypertension predict preoperative correct diagnosis while urinary symptoms increase misdiagnosis risk

**Figure 5:**
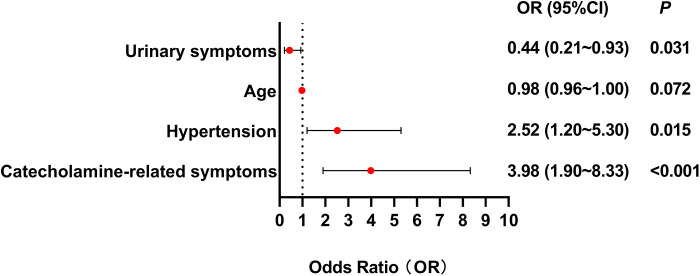
Forest plot illustrating clinical predictors for preoperative correct diagnosis in BPG patients. This forest plot illustrates that catecholamine-related symptoms and hypertension predict preoperative correct diagnosis while urinary symptoms increase misdiagnosis risk. Multivariate logistic regression analysis showed that catecholamine-related symptoms were the strongest predictor of preoperative correct diagnosis (OR = 3.98, 95% CI: 1.90–8.33, *p* < 0.001), followed by hypertension (OR = 2.52, 95% CI: 1.20–5.30, *p* = 0.015). Urinary symptoms were negatively associated with correct diagnosis (OR = 0.44, 95% CI: 0.21–0.93, *p* = 0.031).

Age, tumor size, hypertension, catecholamine-related symptoms, hematuria were found to be significantly related to the correct preoperative diagnosis in univariate logistic regression analysis. Multivariate logistic regression analysis showed that catecholamine-related symptoms were the strongest predictor of preoperative correct diagnosis (OR = 3.98, 95% CI: 1.90–8.33, *p* < 0.001), followed by hypertension (OR = 2.52, 95% CI: 1.20–5.30, *p* = 0.015). Urinary symptoms were negatively associated with correct diagnosis (OR = 0.44, 95% CI: 0.21–0.93, *p* = 0.031). Sex, age and tumor size were not statistically significant (Figure 5).

## 4 Discussion

Bladder paraganglioma (BPG) is an uncommon neuroendocrine tumor that often escapes timely diagnosis due to its rarity and non-specific presentation (3). In this systematic review of 199 reported cases over the past 25 years, we investigated clinical characteristics associated with diagnostic accuracy and examined how early recognition affects surgical outcomes. Our study showed that more than half of BPG patients lack accurate preoperative diagnoses. Patients with preoperative correct diagnosis more frequently received α-adrenergic blockade and underwent cystectomy rather than TUR, with reduced hypertensive crises, surgery discontinuation, and residual tumors compared with those misdiagnosed. Unlike prior reviews limited to descriptive analyses (3, 10), this study proposes a practical, structured diagnostic flowchart aimed at improving clinical decision-making.

Our findings showed that only 40.2% of BPG cases were correctly diagnosed before surgery, consistent with previous reports (10). An in-depth analysis of presenting symptoms revealed that catecholamine-related symptoms can aid diagnosis, whereas urinary symptoms may contribute to misdiagnosis; however, adrenergic manifestations are frequently overlooked. Symptoms of BPG are frequently overlooked by both clinicians and patients, highlighting the need for heightened clinical suspicion. Although 74 patients reported adrenergic manifestations, approximately one-third (26 patients, 35.1%) were misdiagnosed preoperatively, notably, most misdiagnosed patients (22 out of 26) presented with micturition-related symptoms. Given the large sample size, it appears that BPG is often not considered in the differential diagnosis of bladder masses, partly because it is exceedingly rare—accounting for only 0.05% of all bladder tumors—whereas urothelial carcinoma accounts for more than 90% (13). This disparity in prevalence contributes to BPG being overlooked. Multivariate logistic regression analysis showed that catecholamine-related symptoms were the strongest predictor for preoperative diagnosis of BPG (OR = 3.98, 95% CI: 1.90–8.33, *p* < 0.001), the results are in line with those of others (14). It is important to emphasize that adrenergic manifestations like micturition attacks and syncope are often unrecognized or unassociated with bladder lesions by both patients and physicians, as illustrated in the case report by Li S (15).

Intraoperative hypertensive crises frequently occur unexpectedly and demand prompt, coordinated management between urologists and anesthesiologists. Our 25-year retrospective review revealed a 31.2% incidence of intraoperative hypertensive crises, lower than the 42% reported by Uslar et al. in a broader four-decade study of PPGL surgeries (16). Of the 199 patients, 122 (61.3%) had functional BPGs, with approximately 39 patients showing no adrenergic manifestations during routine activities but developing hypertensive crises intraoperatively. Additionally, 11 patients experienced hypertensive crises despite preoperative preparation. Paragangliomas may induce reversible catecholamine-mediated cardiomyopathy and tachyarrhythmias, with adrenergic cardiotoxicity causing acute and chronic myocardial damage (17, 18). In our cohort, five patients who developed intraoperative hypertensive crises experienced cardiovascular complications, all of whom belonged to the misdiagnosed group. This underscores the critical importance of intraoperative communication; To minimize myocardial injury, perioperative prevention should be prioritized. Some authors recommend postoperative monitoring of troponin I in bladder paraganglioma patients, as elevated levels independently predict mortality (19).

Adequate preoperative preparation and meticulous intraoperative management are key to a stable surgical outcome. BPG patients may experience potentially life-threatening hypertensive crises and complications during surgical procedures. Therefore, an appropriate multidisciplinary preoperative evaluation is crucial. This should include comprehensive biochemical, radiological, and cardiac assessments, combined with preoperative *α*-adrenergic blockade therapy and adequate volume expansion before surgery. Such measures can significantly reduce the risk of perioperative complications. Patients diagnosed with BPG before surgery should be prioritized for partial cystectomy, which can reduce surgical interruption and tumor residue.

The difference in surgical outcomes between correctly diagnosed and misdiagnosed patients was striking. Those without a preoperative correct diagnosis were significantly more likely to undergo TUR, experience intraoperative hypertensive crises, require reoperation, or have residual tumor. TUR was performed in nearly 80 percent of misdiagnosed patients, compared to only 15 percent of those correctly diagnosed. While TUR remains a common first-line intervention for bladder masses, it is often inadequate for catecholamine-secreting tumors and may contribute to incomplete resection and tumor recurrence. Previous studies have reported similar concerns regarding oncological control and long-term outcomes following TUR in BPG (20, 21). In contrast, patients who underwent partial cystectomy following a correct diagnosis had fewer complications and more definitive surgical management.

The understanding and classification of PPGL have evolved substantially over recent decades, influencing the WHO taxonomy. The standardization of nomenclature facilitates clearer communication and knowledge exchange among clinicians and researchers. Previously, pheochromocytoma was restricted to intra-adrenal sympathetic paragangliomas, and bladder paragangliomas were considered extra-adrenal pheochromocytomas. However, the current WHO classification defines adrenal pheochromocytoma as paraganglioma of the adrenal gland, and extra-adrenal tumors—including those in the bladder—are classified simply as paragangliomas (22).

**Figure 6:**
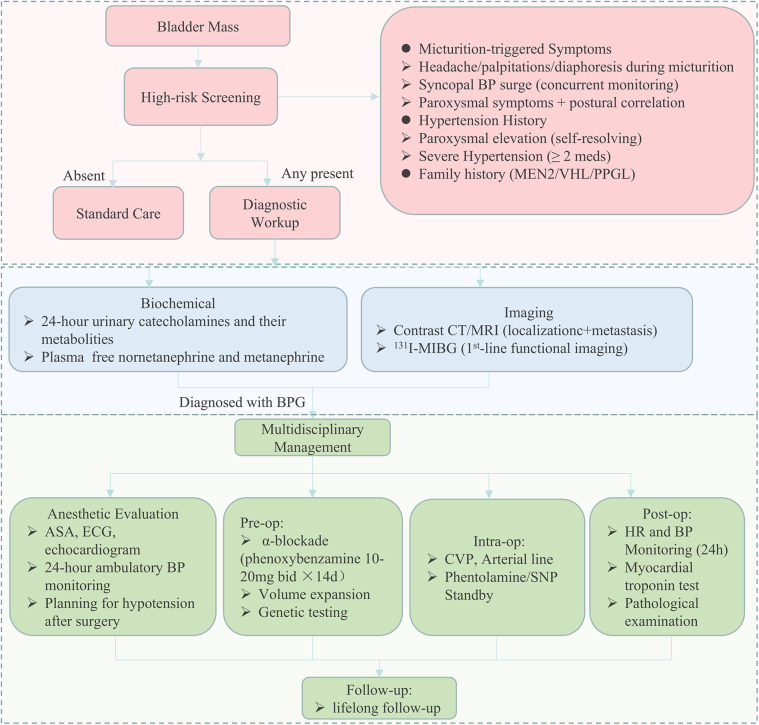
Flowchart for early diagnosis and perioperative multidisciplinary management of bladder paraganglioma. A schematic overview of the recommended diagnostic and perioperative management protocol for patients with suspected bladder paraganglioma. The pathway includes initial clinical assessment of hypertension and micturition-related catecholamine symptoms, followed by biochemical testing and cross-sectional and functional imaging. Preoperative evaluation includes *α*-adrenergic blockade, volume expansion, cardiovascular risk assessment, and genetic testing. Intraoperative and postoperative management measures are also outlined, with an emphasis on hemodynamic monitoring and structured follow-up. ASA, American society of anesthesiologists; CT, computed tomography; MRI, magnetic resonance imaging.

Even though there have been several guidelines on the management of PPGL (23–25), there are few specific guidelines for the diagnosis and management of BPG. That is why we developed an algorithm dedicated to the diagnosis and perioperative multidisciplinary management of BPG. In general, we recommend the “symptom assessment (focus on catecholamine-related/micturition-triggered symptoms) + biochemical testing (plasma/urinary metanephrines/catecholamines) + specific imaging” approach for preoperative BPG diagnosis. This combination leverages symptoms to raise clinical suspicion, biochemical testing to confirm catecholamine activity, and imaging to localize/characterize the tumor—effectively addressing the limitations of single-modal underdiagnosis, as reflected by the lower imaging utilization and symptom recognition in the misdiagnosed group. Our diagnostic algorithm (Figure 6) outlines a stepwise approach based on patient history, blood pressure assessment, catecholamine-related symptom evaluation, and appropriate biochemical and imaging studies. By incorporating common clinical data points, the protocol is readily applicable to routine urological practice. We believe this diagnostic flowchart can be feasibly implemented in urological outpatient settings, where initial clinical contact typically occurs. It offers a concrete strategy to improve early identification of functional bladder tumors and guide appropriate preoperative planning.

Improving clinical recognition of BPG requires targeted efforts at several levels. First, symptom-based screening must be strengthened. Adrenergic manifestations, although subtle, were frequently underrecognized in the misdiagnosed group. Implementing standardized intake questionnaires or history prompts may help capture relevant details early. Second, institutional protocols can assist in bridging the diagnostic gap. Electronic health systems could integrate clinical flags that prompt catecholamine testing or urological referral when certain symptom clusters–such as hematuria with episodic hypertension–are identified. These prompts may be particularly useful for non-specialists who may be less familiar with rare functional tumors. Third, multidisciplinary collaboration should be encouraged. Radiologists, for example, play a critical role in identifying atypical bladder lesions on imaging, while anesthesiologists must be informed in advance to prepare for intraoperative hemodynamic instability. Endocrinologists may assist in biochemical interpretation and preoperative management. Hospitals should consider establishing formal consultation pathways for suspected BPG cases, particularly in referral centers.

## 5 Conclusion

This study demonstrates that delayed or missed diagnosis of BPG remains common and is closely linked to suboptimal surgical outcomes. At the same time, our work provides a pathway for improvement. The diagnostic flowchart we propose is not intended to replace clinical judgment but to offer a structured reference that can support earlier suspicion and safer care. If widely adopted, it may contribute to more accurate diagnoses, fewer complications, and more consistent management of this rare but important condition. This study thereby provides a scientific foundation for establishing clinical guidelines or checklists, which can facilitate early recognition and safer surgical management of BPG by operationalizing the aforementioned multidisciplinary strategies.

## 6 Limitations

This study's strength lies in the large number of BPG cases collected over 25 years with comprehensive data obtained through the PubMed database. However, this study has several limitations. First, as a retrospective analysis, it is inherently subject to selection bias, as the case-report nature may have introduced sampling errors in patient enrollment. Second, incomplete literature retrieval (e.g., only the pubmed database was retrieved) and potential search omissions (e.g., unpublished case reports or language-based restrictions) might have led to the exclusion of relevant studies. Third, heterogeneity in data reporting and study methodologies across included literature (e.g., variable definitions of BPG criteria, inconsistent follow-up durations) complicated the synthesis of uniform evidence. Additionally, the retrospective design precluded the systematic collection of critical variables, such as standardized biopsy protocols and long-term outcome data, which limited the ability to characterize BPG's natural history and treatment efficacy. Furthermore, we acknowledge a potential bias specific to the comparison of imaging efficacy between groups: most patients in the correctly diagnosed group underwent preoperative imaging, while the misdiagnosed group mostly received imaging postoperatively. This difference in the timing of imaging (preoperative vs. postoperative) prevents a fair comparison of imaging sensitivity between the two groups, and may have underestimated the detection rate of the misdiagnosed group—an aspect we have clarified to avoid overinterpreting the diagnostic value of imaging alone. A further limitation is that detailed information on catecholamine evaluation (e.g., specific metabolites tested, plasma vs. urinary samples) could not be extracted due to inconsistent and incomplete reporting in the included literature. This lack of standardized data restricts our ability to clarify the methodology of catecholamine evaluation.

## 7 Future recommendations

Given the limitations, future research should: (1) develop diagnostic scoring systems via multicenter validation; (2) explore AI-based preoperative screening tools for BPG (e.g., machine learning); (3) investigate molecular mechanisms for targeted therapies. Prospective trials are essential to validate these approaches. To improve statistical reliability, the adoption of standardized case-reporting guidelines is warranted, despite challenges in implementation.

## Data Availability

The original contributions presented in the study are included in the article/Supplementary Material, further inquiries can be directed to the corresponding author.
